# Wild Mushrooms: Potential Natural Sources of Antioxidant and Anti-Quorum Sensing Bioactive Compounds for Medical Applications

**DOI:** 10.1155/2023/6141646

**Published:** 2023-10-19

**Authors:** Gebreselema Gebreyohannes, Desta Berhe Sbhatu, Andrew Nyerere, Christine Bii, Abrha Gebreselema Gebrehiwot

**Affiliations:** ^1^Department of Biological and Chemical Engineering, Mekelle Institute of Technology, Mekelle University, Ethiopia; ^2^Department of Medical Microbiology, College of Health Sciences, Jomo Kenyatta University of Agriculture and Technology, Nairobi, Kenya; ^3^Center for Microbiology Research, Kenya Medical Research Institute, Nairobi, Kenya; ^4^Department of Medical Biochemistry, College of Health Sciences, Mekelle University, Ethiopia

## Abstract

**Objective:**

This study was aimed at determining the antioxidant, anti-quorum sensing, and *in vitro* cytotoxic activities of five wild mushroom extracts.

**Methods:**

Wild mushrooms of *Auricularia auricula-judae*, *Termitomyces umkowaani*, *Trametes elegans*, *Trametes versicolor,* and *Microporus xanthopus* were collected from Arabuko-Sokoke and Kakamega National Forests, in Kenya. Specimens were identified and extracted using chloroform (CHL), 70% ethanol (Eth), and hot water (HW) solvents. Antioxidant and cytotoxic activities of the extracts were determined using 2,2-diphenyl-1-picrylhydrazyl (DPPH) and Vero cell lines, respectively, while anti-quorum sensing activities were tested against *Chromobacterium violaceum*. All data were compared using relevant descriptive and inferential statistics at a significance level of *p* ≤ 0.05.

**Results:**

A total of 35 wild mushrooms were collected, identified, and classified into 14 genera. Among screened mycochemicals, fatty acids, flavonoids, polyphenols, and saponins were detected at higher concentrations. The highest free radical scavenging activities of *A. auricula-judae*, *T. umkowaani*, *T. elegans*, and *T. versicolor* were observed in 70% Eth extract with the percentage values of 76.40 ± 0.12%, 68.40 ± 0.01%, 62.40 ± 0.07%, and 66.40 ± 0.04%, respectively, whereas the HW extract of *Microporus xanthopus* showed free radical scavenging activity at 65.90 ± 0.02%. None of the extracts, at the tested concentrations (up to 1000 *µ*g/mL), had shown cytotoxic activity against the Vero cell line. The HW extract of *T. umkowaani* and the 70% Eth extract of *T. versicolor* showed a statistically significant difference in the inhibitory activity of violacein production against *C. violaceum* at the concentration of 200 *µ*g/mL.

**Conclusions:**

The antioxidant activity of wild mushrooms can help to tackle the diseases caused by free radicals. The anti-quorum sensing potential of wild mushrooms could also provide future alternatives to conventional drug therapies cost-effectively. Further detailed chemistry of the bioactive compounds and their possible mechanisms of action responsible for the observed antioxidant and anti-quorum sensing activities are needed.

## 1. Introduction

Mushrooms are primarily underutilized natural resources [1]. Many academics have recently become interested in mushrooms due to their various and distinctive secondary metabolites [2–4]. The possible secondary metabolites of mushrooms have mostly remained unexplored up to this point. Some secondary metabolites, such as carotenoids, anthraquinones, quinolones, oxalic acid, proteins, peptides, phenolics, polyketides, steroids, anthocyanidins, phytoestrogens, terpenoids, phytosterols, glucosinolates, and flavonoids, have only recently been isolated from a small number of mushroom species for their potential therapeutic, nutritional, and medicinal values [5–7]. These metabolites have demonstrated a variety of bioactivities, including hepatoprotective, antiaging, antibiofilm, anticancer, anti-HIV, anti-inflammatory, antimicrobial, antioxidant, antiparasitic, anti-quorum sensing, and antitumor properties [8–17].

Microbial cells use the signal transduction pathway known as quorum sensing (QS) to communicate with one another and within their own species. The QS transduction pathway is started once the signaling molecules have accumulated to a certain concentration and begun to bind to a protein detector. Finally, this route activates the genes that control pathogenicity, rotting, and biofilm formation [18–20]. One of the most effective methods to tackle microbial diseases and antibiotic resistance is the production of anti-quorum sensing agents from natural resources (fungi, bacteria, and plants) [21].

Our bodies manufacture dangerous free radicals as a result of the respiration, digestion, and metabolism process [22]. Free radicals can exist as distinct molecular species with unpaired electrons. Examples of these species include reactive oxygen species, reactive nitrogen species, hydroxyl, DPPH, superoxide anion, hydrogen peroxide, nitrite, and peroxynitrite [23]. Free radicals are extremely reactive and unstable substances that can damage DNA and cell membranes, obstruct the activity of key enzymes and energy production, obstruct numerous cellular processes essential for healthy body operation, and interfere with regular cell division [23, 24].

To the best of our knowledge, no research has been conducted on the detection of antioxidant and anti-quorum sensing activities of wild mushroom extracts obtained from Arabuko-Sokoke and Kakamega National Reserved Forests in Kenya. Therefore, the purpose of this work was to ascertain the antioxidant and anti-quorum sensing capabilities of bioactive compounds isolated from five indigenous Kenyan wild mushrooms.

## 2. Materials and Methods

### 2.1. Description of the Study Areas

**Figure 1:**
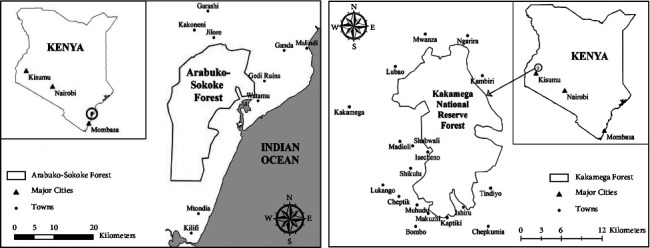
Map of the study areas.

Arabuko-Sokoke and Kakamega National Forests are situated within 3°20′S and 39°55′E and 34°32′0″E and 0°10′15″S, respectively, as shown in Figure 1. Arabuko-Sokoke and Kakamega forests are located in coastal and western Kenya with an average annual rainfall of 900 mm–1,100 mm and 1200–1700 mm, respectively. They are rich in biodiversity and their particular importance gives them a very high conservation value [25].

### 2.2. Study Design

An experimental and exploratory cross-sectional design was used. An experimental (to ensure that the right kind of data can provide a clear and effective response to the research questions) and exploratory cross-sectional design (evaluates both the exposures and the outcome in study participants at the same time) was used.

### 2.3. Sample Collection

During the months of March and April 2018, random collections of wild mushrooms on either tree bark or other substrates (wood, soil, or leaf litter) were made. To prevent breakage and preserve moisture content, specimens were wrapped in aluminum foil and put in an ice box. Molecular and morphological methods were used to identify the specimens, together with the assistance of mycology specialists and relevant literature. Finally, samples were preserved for additional analysis after being dried in an electric drying oven for 480 h at 30°C [26, 27].

### 2.4. Extraction Process of Mycochemicals

Chloroform (CHL), 70% ethanol (Eth), and hot water (HW) solvents were used for the extraction [28, 29]. 100 g of powdered mushroom was combined with 1 L of distilled HW (heated to 60°C for 2 h), 70% Eth (99.9%, Sigma), and 99.8% CHL (Sigma) separately in an Erlenmeyer flask at 25°C and shaken at 150 rpm for 72 h. Whatman No. 1 filter paper was used to filter the extracts, which were then dried in a rotary evaporator at 50°C after being centrifuged at 3000 rpm for 15 min. For additional studies, the extracts were kept in a −80°C deep freezer, freeze-dried, and then kept in a refrigerator at 4°C in an amber-colored bottle.

### 2.5. Qualitative Mycochemical Screening Assay

The extracts were subjected to a qualitative mycochemical screening analysis according to standard procedures [4]. Distilled water was used to dissolve all extracts.

A foam test was used to determine the presence of saponins. 5 mL of distilled water and 1 mL of each 10 mg/mL extract were added, and then the mixture was shaken vigorously. The presence of saponins is indicated by foam production. The ferric chloride (FeCl_3_) test revealed the presence of polyphenols. A solution of 2 mL of distilled water, 3 drops of 10% FeCl_3_, and 3 drops of potassium ferrocyanide were mixed with 1 mL of each extract at a concentration of 10 mg/mL. Blue or green color formation shows the presence of polyphenols [30].

0.5 g of each mushroom extract was boiled in 10% HCl and filtered. To each 2 mL of filtered extracts, 10% ammonia solution was added. The development of pink color in the aqueous layer is a sign that anthraquinones are present [30].

Terpenoids were detected by combining 5 mL of mushroom extracts with 2 mL of CHL, followed by a drop-by-drop H_2_SO_4_ along the tube wall. The presence of terpenoids is shown by the production of brown color at the test tube interface [4].

The tannins were determined by adding 3 mL of 10% FeCl_3_ to each 3 mL of mushroom extract. The presence of tannins is indicated by the production of blue/black color [30].

Mayer's test method was used to determine the alkaloids. Mayer's reagent (1.36 g of mercuric chloride and 5 g of potassium iodide diluted in 100 mL of distilled water) was used to treat a certain amount of the extracts, which resulted in the production of a cream-colored precipitate [4].

H_2_SO_4_ was used to determine flavonoids. A fraction of the extracts were treated with concentrated H_2_SO_4_ and the production of orange color was noticed [30].

Fatty acids were detected by placing 0.5 mL of extract onto filter paper. 0.5 mL of extract at 20 mg/mL was placed on a filter paper. The presence of fatty acids is indicated if the stain on the filter paper persists. A 2 mL of the extract at 20 mg/mL was dissolved in diethyl ether and evaporated to dryness. The presence of volatile oil is indicated by a pleasant odor [4].

### 2.6. Antioxidant Activities of Extracts

The antioxidant properties of *A. auricula-judae*, *M. xanthopus*, *T. umkowaani*, *T. elegans*, and *T. versicolor* extracts in CHL, 70% Eth, and HW were evaluated using the stable free radical 2,2-diphenyl-1-picrylhydrazyl (DPPH) method [28, 29]. In brief, 4 mL of 400 M DPPH was dissolved in dimethyl sulfoxide (DMSO).

In a test tube, various extract concentrations (10, 20, 30, 40, 50, 60, and 70 g/mL) were made, and 1.00 mL of 400 M DPPH was added. When stable absorption values were attained, the mixture was rapidly mixed and left at room temperature for 1 hour in a dark area. As negative controls, 1.00 mL of distilled water, 70% Eth, CHL, and DPPH were made without extracts. A 1.00 mL sample solution was placed in a cuvette and the color changed from purple to yellow and optical density of the solution was measured using a visible spectrophotometer at 517 nm. Different concentrations of ascorbic acid (10, 20, 30, 40, 50, 60, and 70 *µ*g/mL) were used as a standard antioxidant (positive control). Free radical scavenging activity (FRS) of each extract was expressed as the percentage inhibition of the free radical by the extracts, and it was calculated by using the following formula:

(1) $$\begin{array}{c} \%\text{ FRS}=\left[\left(\left(\text{ADPPH}˗\text{AS}\left(\text{AAA}{}^{*}\right)\right)÷\text{ADPPH}\right)\times 100\right], \end{array}$$

where AS is the absorbance of the solution (extract + DPPH), AAA is the absorbance of ascorbic acid, and ADPPH is the absorbance of the DPPH solution.

The extract concentration providing 50% inhibition (IC_50_) was calculated from the graph of RSA percentage against extract concentration [23, 31, 32]. Similar to the antioxidant activty of the extracts calculated above, the standandard antioxidant (ascorbic acid) was calculated as follows:

(2) $$\begin{array}{c} \text{Equation}=\%\text{RSA}=\left[\left(\left(\text{ADPPH}-\text{AAA}\right)÷\text{ADPPH}\right)\times 100\right]. \end{array}$$

### 2.7. *In Vitro* Cytotoxic Activity on Vero Cell Line

The Vero cell line (P171) was grown in T-75 flasks using minimal media that was supplemented with 10% fetal bovine serum (FBS) and 100 g/mL streptomycin (pH 7.25). For 72 h, the T-75 flasks were incubated at 5% CO_2_ and 37°C to reach confluence. Confluent cells were extracted by trypsinization after being rinsed with phosphate buffer saline. After the suspension had been prepared (10 L of cell suspension, 80 L of saline, and 10 L of Trypan blue (0.40%) in a test tube), the number of viable cells was counted using an inverted phase-contrast microscope and the Trypan blue exclusion test on a hemocytometer slide. A 96-well plate was seeded with an aliquot of 2.0 104 cells/mL suspension and incubated in 5% CO_2_ at 37°C for 24 h. Then, in a nutshell, 150 mL of the medium was added to the wells in rows H along with 1,000 g/mL of extracts. From row H to row B, a serial dilution was performed. Row A served as the negative control, and the plates were incubated for 48 h at 37°C with 5% CO_2_. Cell viability was assessed using the 3-(4,5-dimethylthiazol-2-yl)-2,5-diphenyl tetrazolium bromide (MTT) test. The capacity of the living cells to convert the yellow MTT dye into a purple formazan product served as a measure of the cells' growth. 10 L of the MTT dye was added to the cells after 48 h, and they were then incubated for 4 h at 37°C with 5% CO_2_. After removing the entire media from the plates, formazan crystals formed by live cells were dissolved by adding 50 L of dimethyl sulfoxide and vigorously shaking the mixture. Using a microplate reader and a reference wavelength of 720 nm, the absorbance of each well was measured. The following formula was used to calculate cell viability percentage:

(3) $$\begin{array}{c} \left(\left(A-B\right)÷A\right)\times 100, \end{array}$$

where A is the optical density of the control and B is the optical density of extracts. Finally, results were expressed in inhibitory concentration (IC_50_) values. If the IC_50_ is  > 90 *µ*g/mL, the extract is classified as noncytotoxic. If the IC_50_ is between 20 and 89 *µ*g/mL, the extract is classified as moderately cytotoxic. If the IC_50_ is <20 *µ*g/mL, the extract is classified as cytotoxic [33, 34].

### 2.8. Qualitative Anti-Quorum Sensing Activities of Extracts

*Chromobacterium violaceum* (CV026) was cultured in tryptic soy broth (Sigma) with shaking (180 rpm) and incubated at 30°C for 18 h [35]. The overnight culture of CV026 was diluted with sterile distilled water and adjusted to a McFarland standard of 0.5 (1.5 × 10^8^ CFU/ml) using a spectrophotometer. A 100 *μ*L of CV026 and 20 *μ*L of the 100 *μ*g/mL C_6_HSL (hexanoyl homoserine lactone) solution (dissolved in acidified ethyl acetate) were added onto a 5 ml of 1% warm molten tryptic soy agar. Then, the solution was gently mixed and poured immediately over the surface of a solidified tryptic soy agar plate as an overlay. Wells of 5 mm in diameter were made on each plate after the overlay was solidified. A 10 mg/mL extract was dissolved in DMSO and sterilized using a 0.45 *µ*m filter membrane. Of the 10 mg/mL extract, 20 *μ*L, 10 *μ*L, and 5 *μ*L were serially diluted and filled to each well. Another well was filled with 5 *μ*L of 100 *μ*g/mL of C_6_HSL and 45 *μ*L of sterile tryptic soy broth as a negative control. Finally, assay plates were incubated at 30°C for 72 h [36]. The lack of purple pigment around the bacterium was considered an indication of quorum sensing inhibition (inhibition of violacein production). A clear halo zone without bacterial growth (if any) indicates that the extracts have only antimicrobial activity but not anti-quorum sensing activity.

### 2.9. Quantitative Anti-Quorum Sensing Activities of Extracts

*C. violaceum* (20 *µ*L) was inoculated into four Erlenmeyer flasks containing 100 mL lauryl blue (LB) broth and incubated at 30°C for 18 h. The flasks were supplemented with 100 *µ*L of 100 *μ*g/mL C_6_HSL and 20 *µ*L, 10 *µ*L, and 5 *µ*L of extracts from a stock solution (10 mg/mL). The flasks were incubated again at 30°C with shaking (150 rpm) for 20 h. A 1.00 mL of culture was taken from each flask to the test tube and centrifuged at 8,000 rpm for 10 min to precipitate the insoluble violacein. The supernatant was discarded, and then 1.00 mL of DMSO was added to the test tube and shaken for 30 seconds to completely solubilize the precipitated violacein. The test tube was centrifuged at 8,000 rpm for 10 min to remove cells. Then, 200 *µ*L of the supernatant was added to a 96-well plate in triplicates, and absorbance was read at 585 nm. The percentage of violacein inhibition is calculated as

(4) $$\begin{array}{c} \left(\left(\text{Control }{\text{OD}}_{585\text{nm}}\;–\text{ Test }{\text{OD}}_{585\text{nm}}\right)\;÷\;\left(\text{Control }{\text{OD}}_{585\text{nm}}\right)\right)\times 100. \end{array}$$

Simultaneously, the antibacterial effect of the extracts on the growth of the test bacteria was tested by culturing in the presence and absence of extracts and OD was read [37, 38].

### 2.10. Statistical Analysis

The obtained results of each experiment were performed in triplicates and expressed as the mean ± SD values. Microsoft Excel Package was used to analyze quantitative data and draw graphs. All quantitative data were compared using relevant descriptive and inferential statistics at a significance level of *p* ≤ 0.05 [9].

## 3. Results

A total of 35 wild mushrooms were collected from Arabuko-Sokoke National Forest (ASF) (23 specimens) and Kakamega National Forest (KF) (12 specimens). The collected wild mushrooms were classified into 14 genera, namely, *Agaricus*, *Auricularia*, *Boletus*, *Cantharellus*, *Daedaleopsis*, *Ganoderma*, *Lactarius*, *Leucocoprinus*, *Microporus*, *Pleurotus*, *Polyporus*, *Russula*, *Termitomyces*, and *Trametes*. These genera were also classified into edible and inedible mushrooms. The edible mushrooms include *Agaricus*, *Auricularia, Cantharellus, Ganoderma*, *Leucocoprinus*, *Pleurotus*, *Termitomyces*, and *Trametes*. The inedible mushrooms are *Daedaleopsis*, *Microporus*, and *Lactarius*. The wild mushrooms which include both edible and inedible categories are *Boletus*, *Polyporus,* and *Russula*.

### 3.1. Mycochemical Screening

The extracts of the 23 wild mushrooms were qualitatively tested. Alkaloids, anthraquinones, fatty acids, flavonoids, polyphenols, saponins, tannins, terpenoids, and volatile oils were found in various amounts as shown in Table 1. Polyphenol concentrations were highest in *Polyporus* (ASF1) 70% Eth extract, *Trametes* (ASF3) HW and 70% Eth extracts, and *Termitomyces* (ASF14) HW extract. HW, 70% Eth, and CHL extracts of *Termitomyces* (ASF14) yielded moderate quantities of saponins, terpenoids, and fatty acids. Fatty acids and volatile oils were lacking in HW and 70% Eth extracts in all mushroom species.

Different chemical constituents such as flavonoids, polyphenols, saponins, tannins, and terpenoids, among others, were obtained from all extracts as shown in Table 2.

tPolyphenols and flavonoids were found in the highest amounts in the HW extracts of *Termitomyces* spp. (KF2), whereas saponins, tannins, and anthraquinones were found in moderate concentrations. On the other hand, extracts of *Termitomyces* spp. (KF2) in 70% Eth included moderate amounts of tannins, alkaloids, flavonoids, and anthraquinones. Moderate fatty acid concentrations were found in the CHL extracts of *Termitomyces* (KF1), *Termitomyces* (KF3), and *Auricularia* (KF6). The majority of the chemicals lacking from the CHL extracts of most mushroom species were present in the HW and 70% Eth extracts of all mushrooms. All mushroom species' CHL extracts lacked alkaloids or anthraquinones. Similarly, none of the mushroom species' HW and 70% Eth extracts contained any fatty acids or volatile oils.

The most prevalent substances in the HW and 70% Eth extracts of the majority of mushroom species were, in general, polyphenols, tannins, flavonoids, and terpenoids. Different amounts of the mycochemical components of the wild mushrooms were discovered, and they declined in polarity from more polar to less polar solvents. Most substances are soluble in HW and solvents containing 70% Eth, with the exception of fatty acids and volatile oils. The polyphenolic concentration of the aqueous and Eth extracts was higher than that of the CHL extracts.

### 3.2. Determination of Antioxidant Activity of Extracts

#### 3.2.1. FRS Activity of *A. auricula-judae*

**Figure 2:**
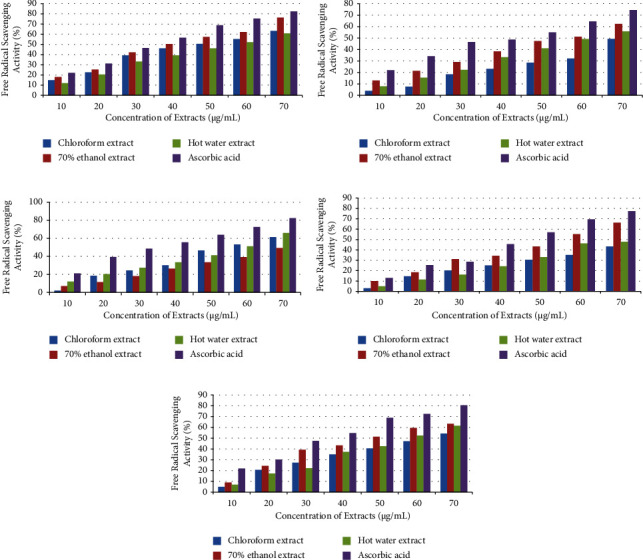
Antioxidant activity of five wild mushroom extracts: (a) *A. auricula-judae*, (b) *M. xanthopus*, (c) *T. umkowaani*, (d) *T. elegans*, and (e) *T. versicolor*.

Each extract of *A. auricula-judae* (KF6) exhibited dose-dependent free radical scavenging (FRS) activity, as shown in Figure 2(a). 70 g/mL was the concentration at which the FRS activity of the extracts in CHL (63.30 ± 0.01%), 70% Eth (76.40 ± 0.12%), and HW (60.90 ± 0.02%) peaked. 40 g/mL, 50 g/mL, and 60 g/mL, respectively, were found to be the inhibitory concentration (IC_50_) values for 70% Eth, CHL, and HW extracts. The ascorbic acid showed the highest FRS activities despite the fact that all the extracts showed good FRS activities.

#### 3.2.2. FRS Activity of *M. xanthopus*

All *M. xanthopus* (KF12) extracts improved FRS activities as extract concentration rose as shown in Figure 2(b). The HW extract had the highest percentage of FRS activity (65.90 ± 0.02%), followed by the CHL extract (61.30 ± 0.01%) and 70% Eth extract (49.40 ± 0.11%). However, the percentage of FRS activity of ascorbic acid (82.40 ± 0.01%) was determined to be significantly higher than that of all other extracts. The IC_50_ values of CHL and HW extracts were determined to be between 50 and 60 g/mL.

#### 3.2.3. FRS Activity of *T. umkowaani*

All *T. umkowaani* (KF2) extracts contained a significant amount of FRS activity as shown in Figure 2(c). Despite the fact that all extracts demonstrated an encouraging proportion of FRS activities, none of them revealed a higher percentage of FRS activities than ascorbic acid (80.40 ± 0.01%). At 70 g/mL, the 70% Eth extract (63.40 ± 0.01%) had the highest percentage of FRS activity, followed by the HW extract (61.50 ± 0.02%) and the CHL extract (54.30 ± 0.01%). The IC_50_ values of CHL extract, 70% Eth extract, and HW extract were 60–70 g/mL, 40–50 g/mL, and 50–60 g/mL, respectively.

#### 3.2.4. FRS Activity of *T. elegans*

The maximal percentage of FRS activity of *T. elegans* (ASF3) extracts in CHL, 70% Eth, and HW was 49.30 ± 0.03%, 62.40 ± 0.07%, and 55.90 ± 0.01%, respectively. When compared to all mushroom extracts, ascorbic acid (positive control) had the highest FRS activity as shown in Figure 2(d). The IC_50_ values for 70% Eth and HW extracts were 50 g/mL–60 g/mL and 60 g/mL–70 g/mL, respectively. However, the CHL extract did not reach the IC_50_ value in the concentrations tested in this study.

#### 3.2.5. FRS Activity of *T. versicolor*

The percentage of FRS activity of *T. versicolor* (KF10) extracts rose as the extract concentration increased from 10 g/mL to 70 g/mL Figure 2(e). In comparison to HW (47.90 ± 0.05%) and CHL (43.30 ± 0.02%) extracts, 70% Eth extract demonstrated the highest percentage of FRS activity (66.40 ± 0.04%) at a concentration of 70 g/mL. The IC_50_ value of 70% Eth was detected at concentrations ranging from 50 g/mL to 60 g/mL; however, the CHL and HW extracts did not achieve the IC_50_ values at the doses examined in this investigation.

### 3.3. Cytotoxic Activity of Five Wild Mushroom Extracts

#### 3.3.1. Cytotoxic Activity of *A. auricula-judae*

**Figure 3:**
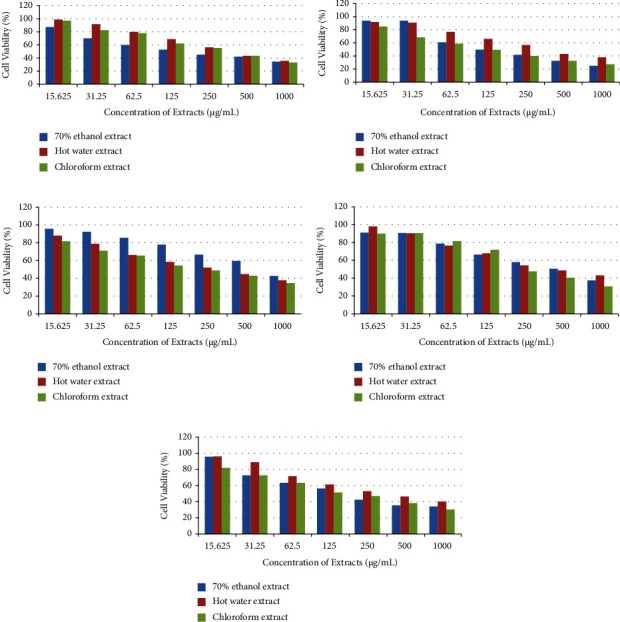
Cytotoxic activity of five wild mushroom extracts: (a) *A. auricula-judae*, (b) *M. xanthopus*, (c) *T. umkowaani*, (d) *T. elegans*, and (e) *T. versicolor*.

Vero cell line cytotoxic activity of *A. auricula-judae* (KF6) extracts was investigated as shown in Figure 3(a). HW extract (98.86 ± 0.03%) showed the highest level of cell viability, followed by extracts made using CHL (97.12 ± 0.01%) and 70% Eth (87.54 ± 0.02%) at a concentration of 15.625 g/mL. The findings showed that all extracts significantly and dose-dependently inhibited the Vero cell line (*p* ≤ 0.05). HW, 70% Eth, and CHL extracts all had IC_50_ values that ranged from 250 to 500 g/mL, 125 to 250 g/mL, and 250 to 500 g/mL, respectively.

#### 3.3.2. Cytotoxic Activity of *M. xanthopus*

The cytotoxic activity of *M. xanthopus* (KF12) extracts showed no significant effect against the Vero cell line as shown in Figure 3(b). The highest percentage of cell viability in CHL, 70% Eth, and HW extracts was detected in 87.51 ± 0.04%, 96.29 ± 0.05%, and 93.19 ± 0.04%, respectively, at a concentration of 15.625 *µ*g/mL. The IC_50_ values of CHL, 70% Eth, and HW extracts were observed at 125 *µ*g/mL–250 *µ*g/mL, 62.5 *µ*g/m–125 *µ*g/mL, and 250 *µ*g/mL–500 *µ*g/mL, respectively.

#### 3.3.3. Cytotoxic Activity of *T. umkowaani*

The extracts of *T. umkowaani* (KF2) demonstrated a dose-dependent cytotoxic effect on the Vero cell line as shown in Figure 3(c). The maximum percentage of cell viability in CHL extract (82.12 ± 0.02%), 70% Eth extract (96.14 ± 0.04%), and HW extract (88.39 ± 0.01%) was observed at 15.625 *µ*g/mL. The IC_50_ values of CHL, 70% Eth, and HW extracts were determined at 125 *µ*g/mL–250 *µ*g/mL, 500 *µ*g/mL–1000 *µ*g/mL, and 250 *µ*g/mL–500 *µ*g/mL, respectively.

#### 3.3.4. Cytotoxic Activity of *T. elegans*

The cytotoxic activity of the three extracts of *T. elegans* (ASF3) was detected in a very low effect against the Vero cell line as shown in Figure 3(d). The IC_50_ values of CHL, 70% Eth, and HW extracts showed within the range of 125 *µ*g/mL–250 *µ*g/mL, 500 *µ*g/mL–1000 *µ*g/mL, and 250 *µ*g/mL–500 *µ*g/mL, respectively. The maximum percentage of cell viability at 15.625 *µ*g/mL was observed in HW extract (98.65 ± 0.02%) followed by a CHL extract (91.53 ± 0.03%) and 70% Eth extract (90.68 ± 0.01%). The pattern indicates that as the concentration of the extracts increases, the viability of the cells decreases.

#### 3.3.5. Cytotoxic Activity of *T. versicolor*

The three *T. versicolor* (KF10) extracts showed a very minimal cytotoxic effect against the Vero cell line as shown in Figure 3(e). The CHL extract (81.74 ± 0.03%), 70% Eth extract (96.52 ± 0.03%), and HW extract (95.96 ± 0.05%) showed the highest cell viability of the Vero cell at 15.625 *µ*g/mL. The IC_50_ values of CHL, 70% Eth, and HW extracts were detected at 125 *µ*g/mL–250 *µ*g/mL, 125 *µ*g/mL–250 *µ*g/mL, and 250 *µ*g/mL–500 *µ*g/mL, respectively.

## 4. Anti-Quorum Sensing Activity of the Mushroom Extracts

### 4.1. Qualitative Anti-Quorum Sensing Activity of the Extracts

**Figure 4:**
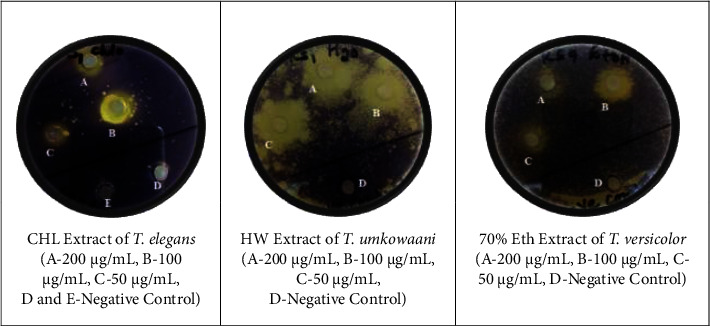
Anti-quorum sensing activity of three mushroom extracts.

All three extracts screened for anti-QS activity inhibited violacein production in a concentration-dependent pattern as shown in Figure 4. The violacein production was inhibited without any antibacterial effect of the extracts on the growth of the bacterium. This was proven by detecting the presence of the same viable cell counts in the extracts treated group and the control group after subculturing onto agar media.

### 4.2. Quantification of Violacein Inhibition of *Chromobacterium violaceum* (CV026)

The HW extract of *T. umkowaani* (KF2) showed a maximum zone of inhibition (14.00 ± 0.04 mm) against the CV026 at the concentration of 200 *µ*g/mL as shown in Table 3. The halo zone (white zone) around the vicinity of the agar wells was a good indicator of the inhibition of violacein production by the CV026. CHL extract of *T. elegans* (ASF3) and 70% Eth extract of *T. versicolor* (KF10), at a concentration of 200 mg/mL, also reduced the production of violacein 11.00 ± 0.05 mm and 11.00 ± 0.01 mm, respectively.

All three extracts showed anti-QS activity at sub-MIC concentrations as shown in Table 4. The percentage inhibitory effect on the production of violacein was reduced by the CHL extract of *T. elegans* (75 ± 0.01%), HW extract of *T. umkowaani* (80 ± 0.02%), and 70% Eth extract of *T. versicolor* (65 ± 0.03%) at 200 *µ*g/mL. The violacein production was reduced as the concentrations of extracts increased. It was observed that the HW extract of *T. umkowaani* and the 70% Eth extract of *T. versicolor* showed a statistically significant difference in the inhibition of violacein production against CV026 (*df* = 2, *F* = 11.08, *p* ≤ 0.05) at 200 *µ*g/mL.

## 5. Discussion

All of the mushroom extracts in the current investigation had significant FRS activities in doses ranging from 10 g/mL to 70 g/mL. On the other hand, a prior investigation found that the FRS activity of mushroom extracts was 2.11 mg/mL [39]. The existence of numerous different secondary metabolites and differences in the quantity and potency of phenolic compounds are likely to be the primary causes of the inconsistent results of the free radical scavenging activities between the current report and earlier ones [40–42]. Numerous other investigations also claimed that mushroom extracts with high phenolic component concentrations showed excellent FRS actions [43–45].

The results of this study show that the FRS activities among the five mushroom extracts varied widely. In this regard, the IC_50_ values of the HW extracts of *A. auricula-judae*, *M. xanthopus*, *T. umkowaani*, and *T. elegans* were observed within the concentration range of 50 g/mL–70 g/mL, with the exception of *T. versicolor*. In contrast to the results of earlier investigations, the current results' IC_50_ values were observed at relatively low extract concentrations. For example, many researchers have observed IC_50_ values of mushroom extracts at 8.68 mg/mL [46], 340 *µ*g/mL [47], and 20.02–0.68 mg/mL [48]. These significant differences could be explained by the presence of reductones, such as ascorbic acid, the number of phenolic compounds, the chemical complexity, and the chemical makeup of the extracts [49]. In addition, different species may have different amounts of the antioxidant components [49, 50].

The most active FRS was found in the *T. versicolor* 70% Eth extract. This may be because the extract in question contains a variety of powerful antioxidant chemicals, which may work in concert to increase the FRS activity. According to one study report, different solvents may have different effects on the quality, strength, and quantity of antioxidant components [51]. The presence of a high concentration of phenolic acids, carotenoids, tocopherols (vitamin E), and ascorbic acids has also been linked to the antioxidant properties of mushroom extracts, according to numerous research [52–55].

Significant cytotoxic activity against the Vero cell line was shown by all five mushroom species' extracts. Crude extracts having IC_50_ values larger than 20 g/mL are regarded as noncytotoxic, according to the American National Cancer Institute (ANCI) recommendations [56]. The IC_50_ values of all five wild mushroom extracts were found to be within the range of noncytotoxic activity. The inhibitory concentrations (IC_50_) of the three extracts from each wild mushroom species differed against the Vero cell line. The discrepancies in cytotoxic activity among mushroom extracts may be attributed in part to changes in compound polarity and solubility, bioactive component modes of action, and the presence of various cytotoxic chemicals in each extract [57]. In contrast to our findings, a prior investigation found that water extracts had extremely low cytotoxic activity on Vero cell proliferation [58]. This variation could be attributable to the geographical location and extraction procedures used in the current and earlier investigations. More research is needed, however, to determine the causes for the discrepancies and their mechanisms of action.

The results demonstrate that the CHL and 70% Eth extracts have higher cytotoxic activity than the HW extract. The existence of numerous and similar chemicals inside the extracts could explain why the CHL and 70% Eth extracts performed better. *T. versicolor* polysaccharides, particularly *β*-glucans, were found to be the most effective antitumor agents [59]. Water-insoluble and alkali-soluble polysaccharides, on the other hand, were shown to have little or no anticancer activity [60, 61].

The anti-QS activity of the *T. umkowaani* HW extract, the *T. elegans* CHL extract, and the *T. versicolor* 70% Eth extract was qualitatively evaluated against CV026. There are no prior publications to compare this work with, as it is the first to demonstrate the anti-QS efficacy of the mentioned mushroom extracts against CV026.

The anti-QS effect of the extracts may result from their ability to limit the production of acyl-homoserine lactones (AHLs), to degrade AHLs, or to block the actions of the autoinducers, despite the fact that their mode of action has not been thoroughly explored. In addition, the CV026's QS system may be interfered with and the genes involved in the quorum sensing process may be downregulated as a result of the synergistic effects of the bioactive substances found in the extracts. According to earlier research, biological quorum quenchers typically disrupt the bacterial quorum sensing circuits by inhibiting the production, transmission, and reception of AHLs signals as well as their receptors with antagonist molecules that are structurally related to AHLs competitive inhibitors [62, 63].

## 6. Conclusion

Fatty acids, flavonoids, volatile oils, polyphenols, and saponins, which have potential uses in nutrition and medicine, were found in considerable concentrations among the mycochemicals that underwent qualitative screening. According to the present findings, mushroom extracts could be good candidates for anti-quorum sensing and antioxidant agents. The mushroom extracts demonstrated very little cytotoxic activity against the Vero cell line indicating that the mushrooms are safe for use in future downstream processes for drug development. The synthesis of the pigment violacein in *C. violaceum* was significantly reduced as a result of the mushroom extracts' anti-quorum sensing abilities. The development of novel alternatives for quorum sensing disruption is attracted by the extracts' ability to quench the quorum sensing activity of the bacterium. Untapped mushrooms' anti-quorum sensing capabilities may add a new dimension to research into the creation of inventive anti-quorum sensing agents that could one day offer cost-effective alternatives to the current pharmacological regimens. The bioactive ingredients that are responsible for the antioxidant and anti-quorum sensing actions need to be identified through additional research. The bioactive substances' precise chemical makeup and any potential mechanisms underlying the observed antioxidant and anti-quorum sensing properties also require a further research.

## Figures and Tables

**Table 1:** Qualitatively screened mycochemicals of wild mushrooms collected from the Arabuko-Sokoke National Forest.

Mushroom species	Code	Mycochemicals screened from three solvents																										
		HW extract									70% Eth extract									CHL extract								
		Saponins	Polyphenols	Anthraquinones	Terpenoids	Tannins	Alkaloids	Flavonoids	Fatty acid	Volatile oils	Saponins	Polyphenols	Anthraquinones	Terpenoids	Tannins	Alkaloids	Flavonoids	Fatty acid	Volatile oils	Saponins	Polyphenols	Anthraquinones	Terpenoids	Tannins	Alkaloids	Flavonoids	Fatty acid	Volatile oils
*Polyporus* spp.	ASF1	−	++	−	+	+	−	++	−	−	−	+++	+	++	−	+	−	−	−	−	++	−	+	−	−	+++	++	+
*Daedaleopsis* spp.	ASF2	+	+	−	+	+	+	+	−	−	+	−	−	+	+	−	+	−	−	−	+	−	−	−	−	−	+	−
*Trametes* spp.	ASF3	−	+++	+	−	++	−	+	−	−	++	+++	−	+	++	−	−	−	−	−	−	−	−	−	−	++	−	+
*Auricularia* spp.	ASF4	++	+	−	−	−	−	−	−	−	+	−	−	++	−	−	+	−	−	−	−	−	−	−	−	+	+	−
*Microporous* spp.	ASF5	−	++	+	−	−	−	−	−	−	−	−	−	+	−	−	−	−	−	−	+	−	+	−	−	−	−	+
*Lactarius* spp.	ASF6	+	+	−	−	−	−	+	−	−	+	−	−	+	−	+	−	−	−	−	+	−	+	−	−	−	+	−
*Russula* spp.	ASF7	−	+	+	+	+	−	−	−	−	−	−	+	−	−	−	+	−	−	−	+	−	−	−	−	−	−	+
*Ganoderma* spp.	ASF8	+	+	+	+	+	+	+	−	−	+	−	+	+	−	+	−	−	−	+	−	−	−	−	−	−	++	+
*Termitomyces* spp.	ASF9	−	−	−	+	−	+	−	−	−	−	−	−	+	+	−	−	−	−	+	−	−	−	−	−	−	−	+
*Pleurotus* spp.	ASF10	+	+	+	+	−	−	+	−	−	−	−	−	−	−	+	+	−	−	−	+	−	−	−	−	−	−	−
*Cantharellus* spp.	ASF11	−	−	−	+	+	−	−	−	−	+	−	+	−	+	+	−	−	−	−	+	−	+	−	−	−	+	−
*Russula* spp.	ASF12	+	−	−	+	+	+	−	−	−	+	−	−	−	+	−	−	−	−	−	−	−	−	+	−	−	+	+
*Termitomyces* spp.	ASF13	−	−	−	−	−	+	−	−	−	−	−	+	+	−	−	−	−	−	−	−	−	−	−	−	+	−	+
*Termitomyces* spp.	ASF14	++	+++	−	−	−	−	−	−	−	+	−	+	++	−	−	−	−	−	−	+	−	−	+	−	+	++	+
*Boletus* spp.	ASF15	−	−	−	−	−	−	+	−	−	−	−	−	+	+	−	−	−	−	−	−	−	−	−	−	−	−	−
*Agaricus* spp.	ASF16	+	+	+	+	+	+	+	−	−	−	−	−	+	−	−	+	−	−	+	−	−	−	+	−	−	+	+
*Leucocoprinus* spp.	ASF17	−	−	+	+	−	−	+	−	−	−	+	−	−	−	+	+	−	−	+	+	−	−	−	−	−	−	−
*Termitomyces* spp.	ASF18	−	+	+	−	−	−	−	−	−	−	−	+	−	−	−	−	−	−	−	+	−	+	−	−	−	−	−
*Termitomyces* spp.	ASF19	−	−	−	−	+	−	−	−	−	+	−	−	−	+	+	−	−	−	−	−	−	−	−	−	+	+	−
*Agaricus* spp.	ASF20	+	−	−	−	+	+	−	−	−	−	+	+	+	−	−	−	−	−	+	+	−	+	+	−	+	+	−
*Agaricus* spp.	ASF21	−	−	−	+	−	+	+	−	−	+	−	+	−	−	−	−	−	−	−	+	−	−	−	−	+	−	+
*Cantharellus* spp.	ASF22	−	−	−	−	+	−	−	−	−	+	+	−	−	+	+	−	−	+	−	+	−	−	+	−	−	+	+
*Cantharellus* spp.	ASF23	+	+	−	−	−	+	+	−	−	−	++	+	−	−	+	−	−	−	+	+	−	−	−	−	−	−	+

−, absence of mycochemicals; +, presence of less concentrations of mycochemicals; ++, presence of moderate concentrations of mycochemicals; +++, presence of high concentrations of mycochemicals.

**Table 2:** Qualitatively screened mycochemicals of wild mushrooms collected from Kakamega National Forest.

Mushroom species	Code	Mycochemicals screened in three solvents																										
		HW extract									70% Eth extract									CHL extract								
		Saponins	Polyphenols	Anthraquinones	Terpenoids	Tannins	Alkaloids	Flavonoids	Fatty acid	Volatile oils	Saponins	Polyphenols	Anthraquinones	Terpenoids	Tannins	Alkaloids	Flavonoids	Fatty acid	Volatile oils	Saponins	Polyphenols	Anthraquinones	Terpenoids	Tannins	Alkaloids	Flavonoids	Fatty acid	Volatile oils
*Termitomyces* spp.	KF1	+	+++	−	+	++	++	+	−	−	−	++	−	−	+	−	++	−	−	−	+	−	+	+	−	++	++	++
*Termitomyces* spp.	KF2	++	+++	++	+	++	−	+++	−	−	+	+	++	+	++	++	++	−	−	−	++	−	+	−	−	−	+++	++
*Termitomyces* spp.	KF3	+	++	+	−	−	++	++	−	−	−	++	−	++	+	++	−	−	−	−	−	−	−	−	−	++	++	−
*Termitomyces* spp.	KF4	+++	−	−	++	+	−	−	−	−	−	+	−	+	−	+	+	−	−	−	−	−	−	−	−	+	−	−
*Auricularia* spp.	KF5	−	−	+	+	−	−	−	−	−	−	−	−	+	+	+	−	−	−	−	+	−	+	−	−	−	−	+
*Auricularia* spp.	KF6	++	+++	++	−	+	+	++	−	−	++	+++	−	++	+	++	−	−	−	+	+	−	+	−	−	+	++	+
*Auricularia* spp.	KF7	−	++	−	−	+	−	+	−	−	+	+	+	−	−	−	+	−	−	−	+	−	−	−	−	−	+	+
*Auricularia* spp.	KF8	+++	−	−	+	−	+	+	−	−	+	−	−	+	−	−	−	−	−	−	−	−	−	−	−	+	−	−
*Termitomyces* spp.	KF9	+	−	−	+	−	+	+	−	−	−	++	−	+	+	+	+	−	−	+	−	−	−	+	−	−	−	−
*Trametes* spp	KF10	++	+++	+	+	++	−	+	−	−	−	+	−	−	−	+	+	−	−	+	+	−	−	−	−	−	−	−
*Microporus* spp.	KF11	−	+	−	−	+	−	−	−	−	+	++	+	−	+	+	−	−	−	−	+	−	+	−	−	−	−	−
*Microporus* spp.	KF12	+	++	+	++	+	−	+	−	−	++	+	+	+	−	−	−	−	−	−	+	−	−	++	−	++	−	++

−, absence of mycochemicals; +, presence of less concentrations of mycochemicals; ++, presence of moderate concentrations of mycochemicals; +++, presence of high concentrations of mycochemicals.

**Table 3:** Anti-quorum sensing activity of mushroom extracts.

Extracts	Concentration (*µ*g/mL)	Zone of QS inhibition (mm)
CHL extract of *T. elegans*	200	11.00 ± 0.01
	100	9.00 ± 0.02
	50	7.00 ± 0.01

HW extract of *T. umkowaani*	200	14.00 ± 0.04
	100	13.00 ± 0.06
	50	12.00 ± 0.08

70% Eth extract of *T. versicolor*	200	11.00 ± 0.05
	100	9.00 ± 0.03
	50	6.00 ± 0.01

QS, quorum sensing. Values are expressed as mean ± SD.

**Table 4:** Percentage inhibition of production of violacein in *C. violaceum*.

Extracts	Percentage inhibition of production of violacein		
	200 *µ*g/mL	100 *µ*g/mL	50 *µ*g/mL
CHL extract of *T. elegans*	75 ± 0.01	60 ± 0.02	48 ± 0.01
HW extract of *T. umkowaani*	80 ± 0.02^*∗*^	69 ± 0.03	56 ± 0.04
70% Eth extract of *T. versicolor*	65 ± 0.03^*∗*^	54 ± 0.01	43 ± 0.02

All values are presented as percentages of the results from the control and are expressed as mean ± SD of three independent (triplicate wells) experiments. ^*∗*^is expressed as the significant difference among the three mushroom extracts (*p* < 0.05).

## Data Availability

The data used to support the findings of this study are available from the corresponding author upon request.

## Abbreviations

ANCI: American National Cancer Institute
ASF: Arabuko-Sokoke forest
C_6_HSL: Hexanoyl homoserine lactone
CHL: Chloroform
CV: *Chromobacterium violaceum*
DMSO: Dimethyl sulfoxide
DPPH: 2,2-Diphenyl-1-picrylhydrazyl
Eth: Ethanol
FBS: Fetal bovine serum
Fig: Figure
FRS: Free radical scavenging
HW: Hot water
HCl: Hydrochloric acid
IC: Inhibitory concentration
KF: Kakamega forest
LB: Lauryl blue
MIC: Minimum inhibitory concentration
MTT: 3-(4,5-dimethylthiazol-2-yl)-2,5-diphenyl tetrazolium bromide
OD: Optical density
QS: Quorum sensing
rpm: Revolution per minute
SD: Standard deviation
